# Quinine localizes to a non-acidic compartment within the food vacuole of the malaria parasite *Plasmodium falciparum*

**DOI:** 10.1186/1475-2875-11-350

**Published:** 2012-10-22

**Authors:** Elaine B Bohórquez, Michael Chua, Steven R Meshnick

**Affiliations:** 1 3113 Michael Hooker Research Center, Department of Microbiology and Immunology, University of North Carolina at Chapel Hill, Chapel Hill, NC, 27599, USA; 2 6129 Thurston Bowles, Michael Hooker Microscopy Facility, University of North Carolina at Chapel Hill, Campus Box 7248, Chapel Hill, NC, 27599-7248, USA; 3 3301 Michael Hooker Research Center, Department of Epidemiology, University of North Carolina at Chapel Hill, Campus Box 7435, Chapel Hill, NC 27599-7435, USA

**Keywords:** Quinine, *Plasmodium falciparum*, *Pfmdr1*, Copy number

## Abstract

**Background:**

The naturally fluorescent compound quinine has long been used to treat malaria infections. Although some evidence suggests that quinine acts in the parasite food vacuole, the mechanism of action of quinine has not yet been resolved. The *Plasmodium falciparum multidrug resistance* (*pfmdr1*) gene encodes a food vacuolar membrane transporter and has been linked with parasite resistance to quinine. The effect of multiple *pfmdr1* copies on the subcellular localization of quinine was explored.

**Methods:**

Fluorescence microscopy was used to evaluate the subcellular localization of quinine in parasites containing different *pfmdr1* copy numbers to determine if copy number of the gene affects drug localization. The acidotropic dye LysoTracker Red was used to label the parasite food vacuole. Time-lapse images were taken to determine quinine localization over time following quinine exposure.

**Results:**

Regardless of *pfmdr1* copy number, quinine overlapped with haemozoin but did not colocalize with LysoTracker Red, which labeled the acidic parasite food vacuole.

**Conclusions:**

Quinine localizes to a non-acidic compartment within the food vacuole possibly haemozoin. *Pfmdr1* copy number does not affect quinine subcellular localization.

## Background

Quinine (QN) was isolated from the bark of the Peruvian Cinchona tree in the mid-1800s [1] and quickly became the treatment of choice for intermittent fever worldwide [2]. Although efficacious, QN use as an anti-malarial requires a relatively long treatment regimen and has substantial side effects. As a result, artemisinin-based combination therapy (ACT) has now been implemented as first-line treatment regimens due to drug efficacy and better patient tolerance. However, QN remains an important treatment option for severe malaria infections [3-5]. Despite the longevity of its use as an anti-malarial, the mechanism of action of QN has not yet been fully resolved.

Early investigations into the anti-malarial activity of QN demonstrated the ability to inhibit chloroquine-induced clumping of haemozoin in the parasite food vacuole [6]. Subsequent evidence found that, like chloroquine, QN interferes with haem crystallization [7,8], indicating that QN acts in the food vacuole. In recent years, parasite resistance to QN has been reported in Africa and Southeast Asia, prompting investigations into resistance mechanisms with a focus on the food vacuole [4,9-13]. Decreased QN sensitivity has been strongly linked to a protein on the food vacuolar membrane, encoded by the *Plasmodium falciparum multidrug resistance* (*pfmdr1*) gene [10-12,14-16]. Whereas some studies have found that *pfmdr1* mutations exert a significant effect on *in vitro* QN sensitivity [10,13,14], other studies have found no association between *pfmdr1* mutations and QN sensitivity [17-19]. Elevated *pfmdr1* gene copy number, however, has been linked to reduced parasite susceptibility to QN in both *in vitro* and clinical studies [12,16,18-20]. Thus, *pfmdr1* has an effect on quinine activity.

Quinine is an aromatic alkaloid that naturally fluoresces under ultraviolet (UV) light. Because of its relatively constant fluorescence quantum yield, QN is commonly used in photochemistry as a fluorescence standard [21]. Surprisingly, the inherent fluorescence of QN has not previously been exploited to investigate its mechanism of action. In this study, the fluorescent properties of QN were exploited to evaluate subcellular localization of the drug by fluorescence microscopy in parasites containing different *pfmdr1* copy numbers in order to determine if copy number of the gene affects drug localization.

## Methods

### Parasite cultivation

*Plasmodium falciparum* cultures of two clonal parasite strains (3D7 and Dd2) with different *pfmdr1* copy numbers and quinine (QN) sensitivity were used in this study. The 3D7 strain has one *pfmdr1* copy and is sensitive to QN. The Dd2 strain has four *pfmdr1* copies [22] and is resistant to QN. Cultures were maintained in candle jars at 37°C under the Trager and Jensen method for malaria parasite culturing [23]. Media was changed every 24 hours. Type O+ serum at 10% and red blood cells (Research Blood Components, LLC: Boston, MA, USA) at 2% haematocrit in malaria culture media (MCM) [24] were used in culturing and experimental conditions.

### Imaging of parasites using fluorescence microscopy

Live asynchronous cultures at 5-8% parasitaemia with 1μM of QN were imaged over an 11-hour period using fluorescence microscopy. Parasitized RBC preparations were plated onto 35mm glass bottom dishes (MatTek, Corp.: Ashland, MA, USA) coated with Poly-L-lysine. A coverslip was carefully applied, and the excess fluid gently squeezed out. The coverslip was sealed with warmed Vaseline to prevent the sample from drying out. Time-lapse imaging of multiple parasites was carried out with a Prior Scientific (Prior Scientific: Rockland, MA, USA) motorized x-y stage. An incubator box maintained the microscope work area including the objective at 37°C. Because *P. falciparum* grows well at 3% oxygen but will tolerate levels as low as 0.5% [25], air was in the incubator box, and the samples were sealed during imaging as stated above. LysoTracker Red DND-99 (Invitrogen: Carlsbad, CA, USA) (50nM) was used to stain the food vacuole and DRAQ5 (Biostatus Limited: Leicestershire, UK) (1μM) was used to stain DNA [26]. Fluorescence images were acquired using an Olympus FV1000 inverted IX81 microscope with a 63x 1.42 NA oil immersion objective at the UNC-Chapel Hill Michael Hooker Microscopy Core Facility. Serial sections with step sizes of 0.20 or 0.25μm were gathered using sequential scanning at 405nm (QN), 559nm (LysoTracker Red), and 635nm (DRAQ5) excitation, and 435±25nm, 595±25nm and 705±50nm emission filters for QN, LysoTracker Red, and DRAQ5 respectively. Confocal pinhole was set to maximum (800μm, ~8 Airy units). Laser transmitted light differential interference contrast (DIC) images were collected at an optimum z-level separately. Image stacks were deconvolved using the iterative blind deconvolution method with Volocity Image Analysis software, version 5.52 (Perkin Elmer: Waltham, MA, USA). Occasionally, Tetraspec 0.1μm beads (Molecular Probes, OR) were added to the sample and used for deconvolution with measured point spread functions. Three-dimensional (3-D) opacity volume rendering and 3-D co-localization analysis was carried out with Volocity.

## Results

### Quinine localizes to the parasite food vacuole

**Figure 1:**
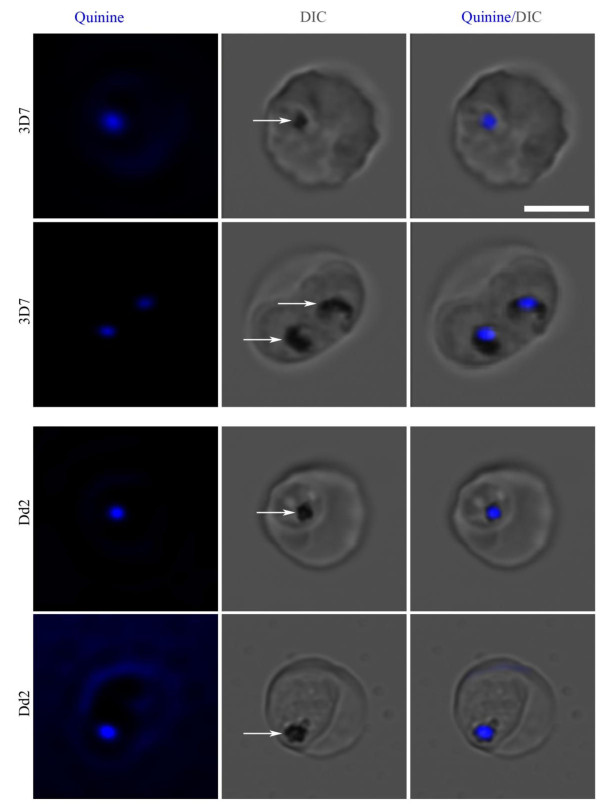
**Quinine localizes with haemozoin in the parasite food vacuole.** Asynchronous parasites of *P*Quinine localizes with haemozoin in the parasite food vacuole. Asynchronous parasites of *P. falciparum* strains 3D7 (one *pfmdr1* copy) and Dd2 (four copies) were incubated with QN to determine its localization. The scale bar represents 5μm. White arrows point to haemozoin crystals in the trophozoite food vacuole. QN (blue) overlapped with haemozoin in both strains and thus localized to the food vacuole.

**Figure 2:**
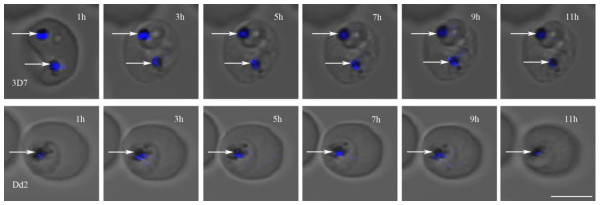
**Quinine localization over time in *P. falciparum*.** Asynchronous parasites were incubated with quinine (blue) to determine subcellular localization of the drug over an 11-hour period. White arrows point to haemozoin crystals in the food vacuole of trophozoites. Quinine overlapped with haemozoin crystals at all time points assessed. The scale bar represents 5μm.

Two-dimensional (2-D) fluorescence and DIC images of both 3D7 (one *pfmdr1* copy) and Dd2 (four *pfmdr1* copies) parasites showed that quinine (QN) co-localizes with haemozoin, which suggests localization in the parasite food vacuole (Figure 1). Similar results were found using a Leica SP2 microscope with a 351nm excitation laser (data not shown). The co-localization was highly repeatable and seen in four fields in at least 10 experiments. Parasites were also imaged at earlier time points following the addition of QN (beginning at one hour of exposure through 11 hours). Quinine fluorescence was found in the food vacuole as early as one hour of exposure and remained there for the duration of the experiment (Figure 2). This effect appears to be independent of *pfmdr1* copy number as experiments in both strains rendered similar results. The same pattern was seen regardless of whether the infected red cells were maintained on the heated microscope stage or in a candle jar, indicating that photobleaching was not a factor. The parasites did not significantly progress through the intraerythrocytic cell cycle during this 11-hour period consistent with a previously observed maturation delay in the intraerythrocytic cell cycle following QN exposure at IC50 and IC99 concentrations for 12 hours [27]. Thus, the apparent halt in cell cycle progression observed in the current study is likely due to the previously reported QN-induced maturation delay phenomenon.

**Figure 3:**
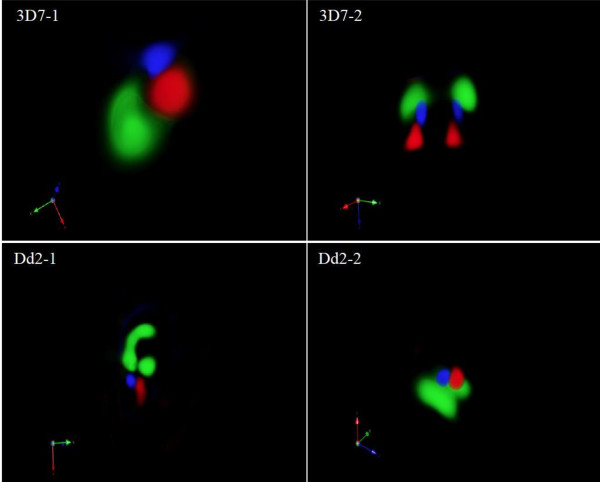
**3-D reconstruction of *P. falciparum *parasites.** Z-stack images of each parasite shown in Figure 1 were used for 3-D volume rendering and colocalization analysis. No QN colocalization with LysoTracker Red was observed in either the 3D7 (left) or the Dd2 (right) strain. Because LysoTracker Red is highly specific for acidic organelles, QN appears to be in a separate and non-acidic compartment of the food vacuole. QN is shown in blue, LysoTracker Red in red, and the DNA stain DRAQ5 in green.

To further characterize subcellular localization of QN, co-localization of QN with either LysoTracker Red, which localizes to acidic compartments, or DRAQ5, which localizes to the nucleus, was investigated using 3-D reconstruction of z-stack fluorescence images. As expected, no co-localization with DRAQ5 was seen. However, QN was found to localize in a separate but adjacent compartment from that in which LysoTracker Red was localized (Figure 3). This 3-D imaging method does not allow for haemozoin to be visualized. The pattern of co-localization was the same for 3D7 and Dd2 parasites, despite their differences in *pfmdr1* copy number. These observations suggest that QN localizes to a distinct, non-acidic compartment within the parasite food vacuole, possibly overlapping with haemozoin in parasites with both low and high *pfmdr1* copy number.

## Discussion

Although QN was the first therapeutic compound used to treat malaria infection [1], its mechanism of action has never been fully resolved [6-8]. Some evidence suggests that parasite resistance to QN is associated with mutations and/or elevated copy number of the *pfmdr1* gene, which encodes for a transporter protein found in the membrane of the parasite food vacuole [9,10,12-14,16,18,20]. Here, the natural fluorescent properties of QN were exploited to obtain insight into the mechanism of action of the drug. Although knowledge of QN’s fluorescent properties has been around since the late-1800s [28], this is the first study to employ the QN’s fluorescence for imaging in the malaria parasite.

Fluorescence microscopy was employed to image QN subcellular localization in two *P. falciparum* strains that contained different *pfmdr1* copy numbers. Quinine consistently overlapped with the haemozoin crystals in both strains when evaluated by 2-D microscopy (Figure 1). However, upon 3-D reconstruction of serial z-stack images, QN was found to reside in a distinct compartment, which is contiguous to, but separate from, the compartment stained by LysoTracker Red. The lack of co-localization with the acidotropic dye suggests that QN resides in a non-acidic compartment within the food vacuole, possibly the same one occupied by haemozoin. This would be consistent with previous reports that quinolone compounds including QN interact with haemozoin crystals directly or with enzymes involved in the haemozoin crystallization process, as previously reported [7,8,29-33]. In summary, these findings suggest that QN is localized in a non-acidic compartment in the food vacuole, possibly that which contains haemozoin.

This study underscores the importance of utilizing the 3-D reconstruction software in imaging studies, since the localization of QN into this novel compartment would not have been detected otherwise. A recent study revealed that QN-haem adducts exhibit fluorescence at least seven-fold greater than QN alone [34]. Thus, it is possible that QN exists in other areas of the food vacuole but cannot be visualized due to the fluorescence intensity of the QN-haem adducts present in these parasites.

Although there was no apparent difference in localization in strains containing different *pfmdr1* copy numbers, the possibility that the *pfmdr1* gene has a role cannot be ruled out. Single nucleotide polymorphisms in *pfmdr1* have also been associated with decreased sensitivity to QN [10,13,14]. Because the protein encoded by *pfmdr1* is a membrane transporter that pumps solutes into the food vacuole, it is possible that mutations within the *pfmdr1* gene could affect the transporter function of the protein by altering the conformation or function of the transporter protein.

In summary, this study is novel because it is the first to exploit quinine fluorescence to study the intracellular distribution of the drug. Here, QN was shown to enter a distinct, non-acidic compartment inside the parasite food vacuole. These results are important because they provide visual support for the hypothesis that QN interferes with haemozoin production, which could guide future studies to investigate a possible interaction between QN and enzymes involved in the haemozoin formation process.

## Conclusions

Quinine localizes to a non-acidic compartment within the food vacuole and *pfmdr1* copy number does not affect QN subcellular localization. These results support previous findings that QN acts in the parasite food vacuole, perhaps interfering with haemozoin formation. This is the first study to provide evidence for QN localization in a sub-compartment of the food vacuole.

## Competing interests

The authors declare that they have no competing interests.

## Authors’ contributions

EBB participated in the design of the study, carried out all of the fluorescence microscopy experiments and data analysis, and drafted the manuscript. MC participated in the acquisition and analysis of microscopy data. SRM conceived of the study and participated in its design. All authors read and approved the final manuscript.

## References

[B1] Bruce-Chwatt LJ Three hundred and fifty years of the peruvian fever bark Br Med J (Clin Res Ed) 1988 296 1486 1487 10.1136/bmj.296.6635.1486 PMC2546010 3134079

[B2] Yeka A Achan J D'Alessandro U Talisuna AO Quinine monotherapy for treating uncomplicated malaria in the era of artemisinin-based combination therapy: an appropriate public health policy? Lancet Infect Dis 2009 9 448 452 10.1016/S1473-3099(09)70109-4 19555904

[B3] WHO World malaria report 2010 World Health Organization, Geneva

[B4] Okombo J Ohuma E Picot S Nzila A Update on genetic markers of quinine resistance in Plasmodium falciparum Mol Biochem Parasitol 2011 177 77 82 10.1016/j.molbiopara.2011.01.012 21295079

[B5] Kyu HH Fernandez E Artemisinin derivatives versus quinine for cerebral malaria in african children: a systematic review Bull World Health Organ 2009 87 896 904 10.2471/BLT.08.060327 20454480 PMC2789363

[B6] Peters W Antimalarial drugs and their actions Postgrad Med J 1973 49 573 583 10.1136/pgmj.49.574.573 4596545 PMC2495604

[B7] Slater AF Cerami A Inhibition by chloroquine of a novel haem polymerase enzyme activity in malaria trophozoites Nature 1992 355 167 169 10.1038/355167a0 1729651

[B8] Egan TJ Ross DC Adams PA Quinoline anti-malarial drugs inhibit spontaneous formation of beta-haematin (malaria pigment) FEBS Lett 1994 352 54 57 10.1016/0014-5793(94)00921-X 7925942

[B9] Zalis MG Pang L Silveira MS Milhous WK Wirth DF Characterization of Plasmodium falciparum isolated from the Amazon region of Brazil: evidence for quinine resistance Am J Trop Med Hyg 1998 58 630 637 9598453 10.4269/ajtmh.1998.58.630

[B10] Sidhu AB Valderramos SG Fidock DA pfmdr1 Mutations contribute to quinine resistance and enhance mefloquine and artemisinin sensitivity in plasmodium falciparum Mol Microbiol 2005 57 913 926 10.1111/j.1365-2958.2005.04729.x 16091034

[B11] Nkrumah LJ Riegelhaupt PM Moura P Johnson DJ Patel J Hayton K Ferdig MT Wellems TE Akabas MH Fidock DA Probing the multifactorial basis of plasmodium falciparum quinine resistance: evidence for a strain-specific contribution of the sodium-proton exchanger PfNHE Mol Biochem Parasitol 2009 165 122 131 10.1016/j.molbiopara.2009.01.011 19428659 PMC3082771

[B12] Sidhu AB Uhlemann AC Valderramos SG Valderramos JC Krishna S Fidock DA Decreasing pfmdr1 copy number in plasmodium falciparum malaria heightens susceptibility to mefloquine, lumefantrine, halofantrine, quinine, and artemisinin J Infect Dis 2006 194 528 535 10.1086/507115 16845638 PMC2978021

[B13] Reed MB Saliba KJ Caruana SR Kirk K Cowman AF Pgh1 Modulates sensitivity and resistance to multiple antimalarials in plasmodium falciparum Nature 2000 403 906 909 10.1038/35002615 10706290

[B14] Mu J Ferdig MT Feng X Joy DA Duan J Furuya T Subramanian G Aravind L Cooper RA Wootton JC Xiong M Su XZ Multiple transporters associated with malaria parasite responses to chloroquine and quinine Mol Microbiol 2003 49 977 989 10.1046/j.1365-2958.2003.03627.x 12890022

[B15] Cowman AF Galatis D Thompson JK Selection for mefloquine resistance in Plasmodium falciparum is linked to amplification of the pfmdr1 gene and cross-resistance to halofantrine and quinine Proc Natl Acad Sci U S A 1994 91 1143 1147 10.1073/pnas.91.3.1143 8302844 PMC521470

[B16] Chaijaroenkul W Wisedpanichkij R Na-Bangchang K Monitoring of in vitro susceptibilities and molecular markers of resistance of plasmodium falciparum isolates from thai-myanmar border to chloroquine, quinine, mefloquine and artesunate Acta Trop 2010 113 190 194 10.1016/j.actatropica.2009.10.016 19879850

[B17] Price RN Uhlemann AC van Vugt M Brockman A Hutagalung R Nair S Nash D Singhasivanon P Anderson TJ Krishna S White NJ Nosten F Molecular and pharmacological determinants of the therapeutic response to artemether-lumefantrine in multidrug-resistant plasmodium falciparum malaria Clin Infect Dis 2006 42 1570 1577 10.1086/503423 16652314 PMC4337983

[B18] Price RN Uhlemann AC Brockman A McGready R Ashley E Phaipun L Patel R Laing K Looareesuwan S White NJ Nosten F Krishna S Mefloquine resistance in plasmodium falciparum and increased pfmdr1 gene copy number Lancet 2004 364 438 447 10.1016/S0140-6736(04)16767-6 15288742 PMC4337987

[B19] Pickard AL Wongsrichanalai C Purfield A Kamwendo D Emery K Zalewski C Kawamoto F Miller RS Meshnick SR Resistance to antimalarials in southeast asia and genetic polymorphisms in pfmdr1 Antimicrob Agents Chemother 2003 47 2418 2423 10.1128/AAC.47.8.2418-2423.2003 12878499 PMC166057

[B20] Anderson TJ Nair S Qin H Singlam S Brockman A Paiphun L Nosten F Are transporter genes other than the chloroquine resistance locus (pfcrt) and multidrug resistance gene (pfmdr) associated with antimalarial drug resistance? Antimicrob Agents Chemother 2005 49 2180 2188 10.1128/AAC.49.6.2180-2188.2005 15917511 PMC1140548

[B21] Fletcher AN Quinine sulfate as a fluorescence quantum yield standard Photochem Photobiol 1969 9 439 444 10.1111/j.1751-1097.1969.tb07311.x 5771430

[B22] Lim P Alker AP Khim N Shah NK Incardona S Doung S Yi P Bouth DM Bouchier C Puijalon OM Meshnick SR Wongsrichanalai C Fandeur T Le Bras J Ringwald P Ariey F Pfmdr1 copy number and arteminisin derivatives combination therapy failure in falciparum malaria in Cambodia Malar J 2009 8 11 10.1186/1475-2875-8-11 19138391 PMC2627910

[B23] Trager W Jensen JB Cultivation of malarial parasites Nature 1978 273 621 622 10.1038/273621a0 351412

[B24] Methods in malaria research 2008 Malaria Research and Reference Reagent Resource Center (MR4)/American Type Culture Collection (ATCC), Manassas, VA

[B25] Scheibel LW Adler A Trager W Tetraethylthiuram disulfide (antabuse) inhibits the human malaria parasite plasmodium falciparum Proc Natl Acad Sci U S A 1979 76 5303 5307 10.1073/pnas.76.10.5303 388434 PMC413130

[B26] Purfield AE Tidwell RR Meshnick SR The diamidine DB75 targets the nucleus of plasmodium falciparum Malar J 2009 8 104 10.1186/1475-2875-8-104 19442305 PMC2689252

[B27] Veiga MI Ferreira PE Schmidt BA Ribacke U Bjorkman A Tichopad A Gil JP Antimalarial exposure delays plasmodium falciparum intra-erythrocytic cycle and drives drug transporter genes expression PLoS One 2010 5 e12408 10.1371/journal.pone.0012408 20811640 PMC2928296

[B28] Urbach F The historical aspects of sunscreens J Photochem Photobiol B 2001 64 99 104 10.1016/S1011-1344(01)00202-0 11744395

[B29] de Villiers KA Gildenhuys J le Roex T Iron(III) protoporphyrin IX complexes of the antimalarial cinchona alkaloids quinine and quinidine ACS Chem Biol 2012 7 666 671 10.1021/cb200528z 22276975

[B30] Pisciotta JM Coppens I Tripathi AK Scholl PF Shuman J Bajad S Shulaev V Sullivan DJ Jr The role of neutral lipid nanospheres in Plasmodium falciparum haem crystallization Biochem J 2007 402 197 204 10.1042/BJ20060986 17044814 PMC1783988

[B31] Leed A DuBay K Ursos LM Sears D De Dios AC Roepe PD Solution structures of antimalarial drug-haem complexes Biochemistry 2002 41 10245 10255 10.1021/bi020195i 12162739

[B32] Egan TJ Ncokazi KK Quinoline antimalarials decrease the rate of beta-hematin formation J Inorg Biochem 2005 99 1532 1539 10.1016/j.jinorgbio.2005.04.013 15927260

[B33] Correa Soares JB Menezes D Vannier-Santos MA Ferreira-Pereira A Almeida GT Venancio TM Verjovski-Almeida S Zishiri VK Kuter D Hunter R Egan TJ Oliveira MF Interference with haemozoin formation represents an important mechanism of schistosomicidal action of antimalarial quinoline methanols PLoS Negl Trop Dis 2009 3 e477 10.1371/journal.pntd.0000477 19597543 PMC2703804

[B34] Alumasa JN Gorka AP Casabianca LB Comstock E de Dios AC Roepe PD The hydroxyl functionality and a rigid proximal N are required for forming a novel non-covalent quinine-haem complex J Inorg Biochem 2011 105 467 475 10.1016/j.jinorgbio.2010.08.011 20864177 PMC3010338

